# Diagnosis of Cholangiocarcinoma: The New Biological and Technological Horizons

**DOI:** 10.3390/diagnostics15081011

**Published:** 2025-04-16

**Authors:** Federico Selvaggi, Loris Riccardo Lopetuso, Andrea delli Pizzi, Eugenia Melchiorre, Marco Murgiano, Alessio Lino Taraschi, Roberto Cotellese, Michele Diana, Marco Vivarelli, Federico Mocchegiani, Teresa Catalano, Gitana Maria Aceto

**Affiliations:** 1 ASL2 Lanciano-Vasto-Chieti, Unit of General Surgery, 66100 Chieti, Italy; 2 Villa Serena Foundation for Research, 65013 Città Sant’Angelo, Italy; r.cotellese@unich.it (R.C.); gitana.aceto@unich.it (G.M.A.); 3 Medicina Interna e Gastroenterologia, CEMAD Centro Malattie dell’Apparato Digerente, Dipartimento di Scienze Mediche e Chirurgiche, Università Cattolica del Sacro Cuore, Fondazione Policlinico Universitario Gemelli IRCCS, 00136 Roma, Italy; lopetusoloris@gmail.com (L.R.L.); marcomurgiano1@gmail.com (M.M.); 4 Dipartimento di Scienze della Vita della Salute e delle Professioni Sanitarie, Università degli Studi Link, 00165 Roma, Italy; 5 Department of Innovative Technologies in Medicine and Dentistry, University “G. d’Annunzio”, 66100 Chieti, Italy; andreadellipizzi@gmail.com; 6 ITAB—Institute for Advanced Biomedical Technologies, University “G. d’Annunzio”, 66100 Chieti, Italy; 7 University “G. d’Annunzio” Chieti-Pescara, Via dei Vestini 31, 66100 Chieti, Italy; eu.melchiorre@gmail.com; 8 Dipartimento di Medicina e Chirurgia Traslazionale, Università Cattolica del Sacro Cuore, 00168 Roma, Italy; 9 Department of Radiology, “Santissima Annunziata” Hospital, 66100 Chieti, Italy; 10 Department of Surgery, University Hospital of Geneva, 1205 Geneva, Switzerland; michele.diana@hcuge.ch; 11 Department of Experimental and Clinical Medicine, Polytechnic University of Marche, 60126 Ancona, Italy; vivarelli63@libero.it (M.V.); federico.mocchegiani@ospedaliriuniti.marche.it (F.M.); 12 Department of Clinical and Experimental Medicine, University of Messina, 98125 Messina, Italy; teresa.catalano@unime.it; 13 Department of Science, University “G. d’Annunzio” Chieti-Pescara, Via dei Vestini 31, 66100 Chieti, Italy

**Keywords:** cholangiocarcinoma, biomarkers, endoscopy, microbiota, liquid biopsy, diagnosis, prognosis, radiomics, radiogenomics, artificial intelligence

## Abstract

The diagnosis of cholangiocarcinoma (CCA) remains challenging. Although new technologies have been developed and validated, their routine use in clinical practice is needed. Conventional cytology obtained during endoscopic retrograde cholangiopancreatography-guided brushings is the first-line technique for the diagnosis of CCA, but it has shown limited sensitivity when combined with endoscopic ultrasound-guided biopsy. Other diagnostic tools have been proposed for the diagnosis of CCA, with their respective advantages and limitations. Cholangioscopy with biopsy or cytology combined with FISH analysis, intraductal biliary ultrasound and confocal laser microscopy have made significant advances in the last decade. More recently, developments in the analytical “omics” sciences have allowed the mapping of the microbiota of patients with CCA, and liquid biopsy with proteomic and extracellular vesicle analysis has allowed the identification of new biomarkers that can be incorporated into the predictive diagnostics. Furthermore, in the preoperative setting, radiomics, radiogenomics and the integrated use of artificial intelligence may provide new useful foundations for integrated diagnosis and personalized therapy for hepatobiliary diseases. This review aims to evaluate the current diagnostic approaches and innovative translational research that can be integrated for the diagnosis of CCA.

## 1. Introduction

Cholangiocarcinoma (CCA) is a tumor that arises from the epithelium of the bile ducts and is the second most common type of liver cancer [1,2,3]. The incidence of CCA is increasing in many countries. The highest rates of CCA are found in northeastern Thailand due to liver fluke infection [2,3,4,5]. In the United States, median overall survival is 8 and 4 months, for patients with extrahepatic and intrahepatic CCA, respectively [6]. CCA is a lethal malignancy due to its biologic aggressiveness, locally advanced disease at presentation and high recurrence rate. The risk factors currently associated with CCA are heterogeneous, all of which can induce cholestasis with chronic inflammation and pathophysiological aspects underlying the development of CCA [7]. Chronic inflammation is responsible for the genesis of CCA by promoting cholangiocyte proliferation [8,9]. Indeed, chronic inflammation leads to immune activation with the release of cytokines and growth factors that induce the recruitment of immune cells and fibroblasts into the tissue microenvironment.

Over time, the microenvironment organizes itself towards deregulated epithelial proliferation that is pro-mutagenic to DNA [10,11]. In bile ducts, chronic inflammation leads to activation of various pathways, including Wnt signaling, and promotes cell proliferation and genetic and epigenetic mutations that predispose to CCA [12]. Although the majority of CCA cases in the West are sporadic, primary sclerosing cholangitis (PSC), hepatolithiasis and parasitic infections are the most common risk factors for the development of CCA [3,4,5,6,13]. Cirrhosis, vital hepatitis, Lynch syndrome, BRCA-associated protein-1 tumor predisposition syndrome, cystic fibrosis and biliary papillomatosis are associated with cancer progression [5,8]. More frequent mutations in the genes *CTNNB1* (1.5%), *AXIN1* (4%) and *APC* (2%) have been described in this tumor [12]. Other hepatobiliary diseases may mimic CCA such as recurrent pyogenic cholangitis, follicular or IgG4 sclerosing cholangitis, eosinophilic or mast cell cholangiopathies, sarcoidosis, ischaemic cholangiopathy, portal hypertensive biliopathy and Mirizzi syndrome [4]. The microbiota has been suggested to play a key role in the progression of PSC and CCA [14]. Mechanisms that may favor the risk of neoplastic development include the release of bacterial toxins and the production of metabolites, modulation of the immune system and inflammation, and metabolic changes in the microbiota and host [15]. The influence of the microbiota in the pathogenesis of CCA now suggests the existence of specific microbial signatures in CCA patients. In a comparison between iCCA, HCC, cirrhosis and healthy controls, a higher abundance of *Firmicutes*, *Bacteroidetes*, *Actinobacteria*, and *Verrucomicrobia* was found in terms of phyla. In addition, CCA patients had the highest a- and b-diversity [16]. Overall, the current diagnostic potential of the gut microbiota in CCA remains to be fully established.

CCA is classified into four subtypes based on location: intrahepatic (iCCA), extrahepatic (eCCA), perihilar/hilar (pCCA) and distal (dCCA) [8,13,17]. According to anatomical location, iCCA arises within the liver parenchyma while pCCA is localized between the second degree bile duct and the junction of the cystic duct into the common bile duct. In dCCA, the tumor is confined to the common bile duct below the cystic duct [3,18]. Nowadays, pCCA is the most common biliary tumor, accounting for 40–60% of patients, followed by dCCA (20–30%) and iCCA (10–20%) [13]. Macroscopically, CCA has four growth patterns: mass-forming, periductal infiltrating, intraductal and mixed [3,13,17]. Biliary intraepithelial neoplasia (BilIN) is a precursor of CCA with a large duct and intraductal papillary neoplasm of the bile duct (IPNB). Although CCA is genomically heterogeneous, distinct genetic alterations have been observed. Specifically, small-bile-duct iCCAs show mutations in *IDH1/2* and *FGFR2* fusions, whereas large-bile-duct iCCAs, pCCAs and dCCAs show *KRAS* and *TP53* genetic alterations [8,19]. *SMAD4* mutations have been reported in 10-20% of cases [4].

The diagnosis of CCA is usually made in an emergency setting, highlighting the late presentation of symptoms [1,4]. Despite advances and new technologies in the management of CCA, the prognosis remains poor [6].

Overall survival after diagnosis of CCA is less than 10% [4,9]. This is due to its biologic aggressiveness, high recurrence rate and advanced stage at presentation [9]. The diagnosis of CCA is complex and multifaceted, due to its asymptomatic nature [13]. Traditional diagnostic modalities include imaging studies with ultrasonography (US), contrast-enhanced computed tomography (CECT), magnetic resonance imaging (MRI) and endoscopic retrograde cholangiopancreatography (ERCP) with biopsy [13]. Unfortunately, these current techniques are often inconclusive with low accuracy [13,20,21]. In current clinical practice, a combination of imaging and endoscopic techniques is required for the diagnosis and staging of CCA [3,13,20,22]. Currently, CECT is the initial standard imaging modality that allows the assessment of CCA and its relationship to vessels and perineural invasion status [3,23]. The accuracy of CECT is greater than 85%, with an estimated sensitivity and specificity of 89% and 92%, respectively [4]. Specifically, MRI with hepatobiliary contrast is the most accurate modality for identifying liver metastases in patients with mass-forming iCCA [3,24,25]. On the other hand, magnetic resonance cholangiopancreatography (MRCP) is indicated for pCCA to determine resectability [23]. In the preoperative clinical setting, the endoscopic ultrasound (EUS) with fine-needle aspiration (FNA) plays a crucial role in the diagnosis of CCA. Its sensitivity is influenced by the location of the tumor. In fact, EUS-FNA is more sensitive for the diagnosis of eCCA (sensitivity range: 43–89%) [6]. The technological evolution of EUS is intraductal ultrasound (IDUS). IDUS consists of a high-frequency (12–30 MHz) miniprobe and is indicated for the examination of the proximal biliary system, portal vein and right hepatic artery. The limitation of IDUS is that it cannot be used for FNA [6]. Three-dimensional models of the biliary tree and quantitative metrics have been developed to overcome the subjectivity of MRCP [26]. 18F-fluoro-deoxy-glucose positron emission tomography (18F-DG-PET) CT is recommended as part of CCA staging because of its utility in detecting nodal and distant metastases [3,13]. Biopsy is mandatory to confirm the diagnosis of CCA, which is ultimately made by immunohistochemical results [1]. CCA is highly positive for cytokeratin (CK) 7 and 19 [4]. Only 20% of CCA cases are documented as CK20-positive. To optimize the differential diagnosis, the complete antibody panel consists of staining for CK7, CK20, hepatocyte paraffin 1, CDX2, TTF-1, arginase-1, glypican-3 and monoclonal carcinoembryonic antigen [6]. A panel of C-reactive protein (CRP), N-cadherin and S100 calcium-binding protein P (S100P) was used to confirm the morphologic subclassification of CCA [27]. After surgical resection with curative intent, only 20–35% of patients have an overall survival of 5 years. Although surgery remains the cornerstone of curative therapy, the incidence of incomplete resection remains high, ranging from 10 to 85% and depending on experience and CCA location [6,16]. Surgical resection is feasible in less than 25% of CCA patients [28]. In the ABC-02 trial, the median overall survival was 11.7 months with the use of gemcitabine and cisplatin combined in unresectable, recurrent and metastatic biliary tract cancer [8,29]. Neoadjuvant chemotherapy is not recommended in patients with resectable CCA. First-line treatment with cisplatin plus gemcitabine in addition to immunotherapy is indicated in cases of advanced biliary tract cancer [4,9,19]. However, it remains to be seen which patients will benefit from immunotherapy, as the difference in median overall survival between durvalumab and gemcitabine–cisplatin is 1.3 months [19]. Unfortunately, the prognosis for advanced CCA remains poor, with only 2–5% of patients surviving 5 years [5,8]. The benefit of chemotherapy is limited to patients with good performance status [9].

In this review, we explore the progress made in CCA diagnosis with emphasis on the new biological and non-biological applications. Among the biological methods, the impact of specific CCA biomarkers, liquid biopsy technology and the microbial signature in CCA patients have been analyzed. The innovative translational research based on artificial intelligence, radiomics and radiogenomics has been critically covered and deepened towards a more precise and standardized diagnostic work-up.

## 2. Biological Approaches to the Diagnosis and Prognosis of CCA

### 2.1. Diagnostic and Prognostic Biomarkers for CCA

The identification of specific biomarkers is crucial for diagnosis, monitoring response to therapy and identifying personalized medicine. Circulating biomarkers include proteins and metabolites released by CCA cells, as well as free-floating DNA and RNA. The biomarkers are detected in bile, blood and tumor tissue. For the clinical diagnosis of CCA, the carbohydrate antigen 19-9 (CA 19-9), carcinoembryonic antigen (CEA) and alpha-fetoprotein (AFP) are routinely used in the differential diagnosis with hepatocellular carcinoma (HCC) [2,6,13,30]. CA 19-9 is an epitope of the Lewis antigen that is produced by biliary duct, salivary, endometrial, gastric and colonic epithelial cells [6]. In CCA, CA 19-9 has a sensitivity of 50–90% and a specificity of 54–98% [3,6]. CA19-9 levels need to be interpreted in the setting of biliary obstruction, and it is important to note that 10% of patients are non-producers and have a normal CA19-9 level [13]. CEA levels (>5.2 ng/mL) have a sensitivity of 68% and a specificity of 82% [6]. Other biomarkers are CK19 fragment (CYFRA 21-1) and CA 242, which showed higher specificity than CA 19-9 in patients with iCCA [8]. In the diagnostic algorithm, matrix metalloproteinase-2 (MMP-2) and tissue inhibitor of metalloproteinase-1 (TIMP-1) are considered to be promising biomarkers that could aid in the differential diagnosis of CCA [2]. It is important to recognize that serum CA 19-9 levels are elevated in other non-malignant conditions such as cholangitis, biliary obstruction and primary biliary cirrhosis [3]. Unfortunately, serum IgG4 is not useful in screening for malignancy, because IgG4 levels can be increased in patients with sclerosing cholangitis as well as in CCA patients [6]. In bile samples, sperm-specific protein 411 (SSP411) and Wisteria floribunda agglutinin (WFA) showed elevated levels in CCA patients, by representing a potential differentiator with benign hepatobiliary disease [8]. In serum, the A1B-Glycoprotein (A1BG)/afamin (AFM) ratio and RB-associated KRAB zinc finger protein (RBAK) exhibit elevated levels in CCA patients compared to benign disease [8]. In bile and tissue samples, tumor type M2 pyruvate kinase (TuM2-PK) is an emerging biomarker used to differentiate CCA from benign lesions. Elevated levels of TuM2-PK are associated with lymph node metastasis [8]. Biomarkers include proteins, circulating tumor DNA and RNA, circulating tumor cells, cytokines and exosomes. The combination of the biomarker panel with the innovative technique of liquid biopsies to identify proteins from the patient’s fluid sample will improve diagnostic accuracy in CCA. Currently, biliary biomarkers are not recommended to replace histologic and cytologic findings [4]. Interestingly, extracellular vesicles (EVs) are potential markers for CCA. Promising and additional serum biomarkers are strongly advocated to achieve a non-invasive and rapid diagnosis of CCA.

### 2.2. Liquid Biopsy for Diagnosis of CCA

The biliary tree is lined by cholangiocytes, which are highly polarized epithelial lining cells that mediate the contents of the luminal bile fluid and transport through signaling pathways influenced by endogenous and exogenous molecules such as bile acids, hormones or neurotransmitters, or microbial products and drugs [31,32]. In the tissue microenvironment, cholangiocytes exert their function through receptor-mediated endocytosis and apical exocytosis of EVs (30–100 nm), while communication between neighboring cholangiocytes may also occur through cytoplasmic signaling pathways [31,33]. Cholangiocytes are able to modulate the intrahepatic microenvironment by diversifying their secretome. Bile duct injury induces changes in cholangiocyte phenotypes and the release of soluble mediators, which may depend on the specific etiology. Cytokines secreted by cholangiocytes drive ductal cell proliferation, inflammation, portal fibrosis and carcinogenesis [34]. The evaluation of qualitative and quantitative biomarkers produced by cholangiocytes in hepatobiliary pathological contexts is of great interest for their utility in diagnostic and prognostic purposes. In the clinical setting, the pathological diagnosis of CCA can be obtained by ERCP using bile duct brushing and forceps biopsies, but the sensitivity of these approaches to detect malignant cells may vary according to the expertise of the hepatobiliary center [3,4,21,35]. Other adjunctive detection techniques for CCA, such as digital image analysis, KRAS mutation testing and multicolor fluorescence in situ hybridization (FISH), can be used, although these methods showed variations in accuracy [21,35]. In this context, there is a strong need for new and more specific diagnostic approaches in the diagnosis, management and monitoring of CCA. The accuracy of current imaging tests and circulating tumor biomarkers for the diagnosis of CCA is far from satisfactory [36]. Moreover, the stealthy growth of the tumor makes it difficult to detect in its early stages, depriving patients of early access to curative treatments [37]. In CCA patients, liquid-biopsy-based analytical applications could facilitate early diagnosis of CCA in at-risk individuals. Through liquid biopsy analytical applications, predictive molecular pathology can evaluate molecular biomarkers of tumor origin from fluid biological samples such as saliva, urine, blood and drainage fluid [38]. The use of liquid biopsies allows the identification of various circulating tumor-derived products such as metabolic marker proteins, circulating tumor cells (CTCs), cell-free DNA (cfDNA), exosomes and microRNA [39]. In particular, CTCs can be detected in the blood of CCA patients. Their increased levels are associated with higher tumor aggressiveness and poor prognosis, and can therefore identify CCA patients at risk of early death [32]. Mutation-based cfDNA analysis is another application used in hepatobiliary research. In CCA, miR-21 and miR-221 were significantly overexpressed in plasma. Increased expression of miR-21 is associated with a poorer prognosis [39]. However, the increased expression of miR-21 and miR-221 is not only detected in CCA but also in HCC and other hepatobiliary diseases [39]. In addition, high levels of CYFRA 21-1, MMP-7, osteoblasts, periostin and IL-6 were detected in the serum of CCA patients, which may be useful for further diagnosis of CCA [39]. Unlike miRNAs and CTCs, which are detected in blood samples, cfDNA can be detected in the bile of CCA patients, and tumor recurrence and prognosis can be inferred primarily by detecting single-nucleotide SNP variants, cfDNA insertions and deletions, but not their expression [40,41]. Genetic profiling of mutations present in CCA can be used in combination with clinical data to identify subgroups of patients who are refractory or unresponsive to treatment. In iCCA, alterations in TP53, KRAS and CDKN2A have been reported as independent prognostic factors in relation to clinical and pathological variables, tumor stage and treatment [42].

Next-generation sequencing is indicated for profiling at diagnosis in CCA and for identifying targetable gene alterations to guide second-line therapy in advanced CCA after first-line treatment with cisplatin plus gemcitabine and durvalumab [43]. Although accurate early diagnostic methods for CCA are not yet available, new approaches based on “-omic” technologies may offer solutions for interpreting the somatic heterogeneity of CCA and its personalized treatment. Indeed, there are numerous variables that can be considered and that could become useful routine biomarkers, such as circulating nucleic acids, proteins, metabolites, EVs and finally CTCs. Recent approaches have searched for protein biomarkers in serum EVs or tumor-cell-derived liquid biopsy for personalized medicine useful for high-risk individuals affected by PSC. Protein biomarkers identified in EVs were validated by ELISA, and their expression was also assessed by RNA single-cell analysis in CCA [36]. Protein- and etiology-based logistic models with predictive, diagnostic or prognostic capabilities that combine multiple circulating protein biomarkers appear to be the most promising method for personalized clinical therapy. Protein families evaluated in this type of analysis include proteases, antioxidants and oncogenic proteins, which may also result from the presence of parasitic microbes and trematodes that promote cholangiocarcinogenesis [44]. Comparative studies of protein biomarkers from serum EVs were performed in patients with CCA, PSC and HCC compared to healthy controls. Proteomic analysis of EVs revealed a higher abundance of oncogenic proteins from tumor cholangiocytes compared to EVs released from normal human cholangiocytes. Similar human oncogenic proteins were found in EVs from mouse serum after orthotopic implantation of human CCA cells [45]. Ultra-performance liquid chromatography–mass spectrometry (UPLC-MS) analyses were used to characterize the urinary metabolic profile of CCA patients. The largest class differences were observed between the acylcarnitine, bile acid and purine metabolic profiles of healthy controls compared to CCA patients, rather than patients with benign biliary strictures. In fact, the latter showed metabolic profiles similar to those of CCA and are therefore not applicable for early diagnosis of CCA [46]. Genomic analyses of mutation, methylation, gene expression and non-coding RNA profiles have shown that iCCA and eCCA are defined as two distinct genetic entities [47]. However, among the most frequently mutated genes in CCA, such as *CDKN2A/B*, *IDH1/2*, *BAP1*, *BRAF*, *ARID1A*, *KRAS*, *TP53*, *TERT*, *SMAD4*, *BRCA1/2*, *TGFBR2*, *RBM10*, *NF1*, *SPTA1*, *RB1*, *KMT2C* and *DDR*, the frequency varies according to geographical origin, patient lifestyle and the design of the mutation analysis [32,48,49]. In particular, dysregulation of non-coding RNAs has been implicated in controlling the expression of genes that regulate cell survival, proliferation and chemoresistance in CCA [32,50]. These biomolecular features represent the current landscape of human CCA diagnostic biomarkers from specifically expressed genes.

### 2.3. Gut Microbiota and CCA

The gut microbiota consists of microbial communities organized in complex structures [51]. It plays a crucial role in digestion, absorption, immune response and metabolism [52]. An important class of microbially produced metabolites are bile acids, a liver product that is metabolized from cholesterol by the gut microbiota and regulates metabolic pathways. In turn, bile acids influence the microbial composition of the gut, creating a dynamic interplay between the host and microbiota [53].

**Figure 1:**
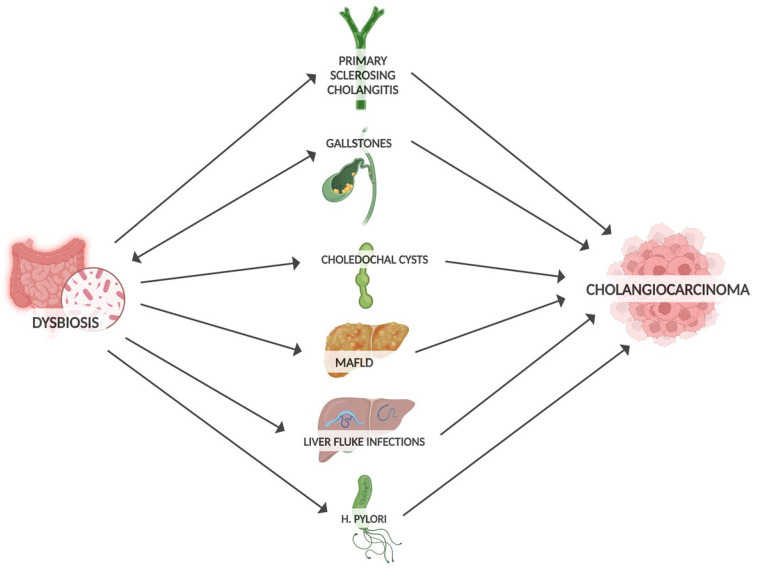
Risk factors for CCA influenced by gut microbiota.

Well-established risk factors for developing CCA include gallstones, PSC, liver infection and biliary obstruction (Figure 1). By influencing these precancer risk factors, dysbiosis may promote CCA progression [54]. Microbiota can promote gallstone formation by regulating bile acid metabolism. Conversely, gallstones may promote intestinal dysbiosis through altered bile secretion and inflammatory response [55]. Large numbers of bacteria have been detected in bile and gallstones [56]. At the phylum level, the bile microbiota composition closely resembles that of the gut microbiota [57]. The widespread application of next-generation sequencing technology has allowed the investigation of the relationship between the gut microbiota and other cancers, including biliary tract cancer [58]. In terms of genera, a predominance of *Pseudomonas*, *Bacillus*, *Enterococcus* and *Acinetobacter* was found in the bile of patients with gallstones [59]. In comparison with controls, the microbiota of these patients showed a decrease in diversity. Notably, an increase in *Oscillospira* levels (which correlates positively with secondary bile acids) and a decrease in *Roseburia* were found [60]. Similarly, an increase in Proteobacteria was observed, while *Faecalibacterium*, *Liachnospira* and *Roseburia* were decreased [57]. PSC is associated with a higher risk of CCA compared to patients without PSC [61]. Patients with PSC also had reduced gut microbiota diversity, with an increased abundance of *Veillonella* [62]. In addition, the genus of *Enterococcus* was positively associated with serum levels of alkaline phosphatase, so it was considered a biomarker of disease activity [63]. In addition to the gut microbiota, the mycobiota is also altered in PSC, with an increased abundance of *Exophiala* and a reduction in *Saccharomyces cerevisiae* in PSC patients [64]. In PSC, dysbiosis also affects the bile. Reduced species diversity of the biliary microbiome has been found in PSC patients, with an increased abundance of *Enterococcus faecalis*, which is positively correlated with levels of the harmful taurolithocholic acid with potential carcinogenic properties [65]. Liver fluke infections, a group 1 carcinogen, are considered one of the risk factors for CCA [7]. An increase in *Lactobacillus* ssp., *Aggregatibacter* spp., *Klebsiella* spp., *Treponema* spp. and *Staphylococcus equorum* has been found in the bile of infected patients [66]. Enrichment of *Bifidobacteriaceae*, *Enterobacteriaceae* and *Enterococcaceae* in liver-fluke-associated bile duct tissue suggests that liver flukes may facilitate the translocation of these bacteria [66,67]. Choledochal cysts are also considered rare precancerous lesions of CCA. Many studies have shown an increased abundance of enterobacteria such as *Escherichia coli* and *Klebsiella*, but also of non-enterobacteria, in the bile of patients with choledochal cysts [68]. A strong association between gut dysbiosis and liver metabolic disease (MAFLD) has been found through an increase in hepatic ferroptosis, a programmed cell death related to iron-dependent lipid peroxidation, which can be enhanced by the bacterial translocation to the liver and which has a key role in the progression of MAFLD [69].

In patients with inflammatory bowel diseases, altered microbiota has been shown to influence the progression of PSC and increase the risk of CCA [70]. One study showed that the dysregulation of the immune system (leading to the CCA onset) is greater in patients with PSC and active colitis than in patients with PSC and inactive colitis or PSC without colitis, suggesting a key role for microbiota in the progression of colitis, PSC and CCA [14]. *Helicobacter pylori*, a gastric-cancer-related bacterium, is also associated with the risk of other types of cancer. Several studies have shown an increase in *Helicobacter* spp. in stool samples from patients infected with *Opisthorchis viverrine*. The *CagA* and *CagE* genes, which encode proteins involved in the phosphorylation of sarcoma family kinases (and consequently involved in the signal pathways of fibrosis and inflammation of the bile ducts), were overexpressed [71]. In vitro experiments showed the ability of CagA-positive *Helicobacter* species to increase levels of the anti-apoptotic factor Bcl2 and activate NF-kB signaling pathways in co-cultured CCA cells, with the proliferation of bile duct cancer cells [72]. When CCA patients were compared with normal counterparts, *H. pylori* was found in the bile and biliary duct tissues of hepatobiliary cancers at increased levels compared to controls [73,74]. *H. pylori* protein seropositivity has also been associated with an increased risk of CCA development [75].

Evidence in the literature suggests a potential role of microbiota for the development and progression of CCA. Dysregulation of the intestinal barrier allows bacterial translocation and contamination of the otherwise physiologically sterile biliary tract (gut–liver axis), resulting in the production of toxins or other substances that can change the composition of bile and can alter the biliary immune system [71]. The link between the biliary tract and gut microbiota in the pathogenesis of CCA has been elucidated in animal models. Specifically, disruption of the barrier function results in the passage of gut bacteria and LPS into the hepatic circulation, via the portal vein, to the liver and biliary tract [76]. From there, polymorphonuclear myeloid-derived suppressor cells (PMN-MDSC) rise via a TLR4-overexpressed pathway (associated with CCA progression and worse disease outcomes) and the CXCL1–CXCR2 axis, promoting chronic inflammation, tumor proliferation and reducing anti-tumor activity [77]. One hypothesis for a pathogenic link between microbiota and CCA is related to MDSCs, which are known to facilitate tumor progression by suppressing cytotoxic T lymphocytes and enhancing angiogenesis, tumor invasion and metastasis [78]. This was demonstrated in a recent study in which the reduced expression of CXCL1 and suppression of PMN-MSDC led to the inhibition of tumor growth in a mouse model [79]. Regarding genes, CCA patients had higher levels of *Alloscardovia*, *Peptostreptococcaceae*, *Actinomyces* and *Lactobacillus* [16]. Similarly, specific gut-microbiota-based signatures, including the genera *Faecalibacterium*, *Klebsiella*, *Ruminococcus Gnavus group*, *Lactobacillus*, *Dorea*, *Veillonella*, *Burkholderia*, *Caballeronia*, *Paraburkholderia* and *Citrobacter*, have been demonstrated to distinguish CCA and HCC (Table 1) [80].

A scoping review based on 12 studies and one systemic review analyzed the correlation between the gut microbiota and the diagnosis, prognosis and progression of biliary tract cancer. The included studies reported lower abundances of the genera *Faecalibacterium*, *Ruminococcus*, *Megamonas*, *Burkholderia*, *Caballeronia* and *Paraburkholderia*. Conversely, higher abundances of the genera *Actinomyces*, *Alloscardovia*, *Lactobacillus*, *Bacteroides*, *Alistipes*, *Shigella* and *Klebsiella* were reported [81].

Cholangiocytes are regularly exposed to a variety of commensal microbes and their molecules, which can have a significant impact on the homeostasis of the cholangiocyte microenvironment. The bile microbiome of CCA also had specific microbial signatures. Analysis of the bile microbiome of PCC patients revealed an increase in *Enterococcus faecalis*, *Enterococcus*, *Enterobacter cloacae* and *Escherichia coli* as the most common bacterial species [82]. In a comparison between patients with pCCA and patients with gallstones, *Pyramidobacter*, *Klebsiella*, *Bacteroides* and *Enterococcus* were found to be the most dominant bacteria in the bile of patients with pCCA [83]. These findings were supported by another study comparing the biliary microbiota of eCCA patients with that of individuals with cholelithiasis, which showed a higher abundance of the genera *Bacteroides*, *Geobacillus*, *Meiothermus* and *Anoxybacillus* in eCCA patients [84]. Specific microbial signatures have also been observed in the blood microbiome of CCA patients. At the phylum level, the most dominant bacteria were Cyanobacteria, Bacteroidetes, Actinobacteria, Firmicutes and Proteobacteria [85]. In addition, the gut microbiota has also been associated with treatment outcomes in metastatic or unresectable iCCA. Seventeen patients, under chemotherapy (combination of cisplatin and gemcitabine) were enrolled and divided into two groups: chemotherapy responders and non-responders. The taxa *Ruminococcaceae*, *Oribacterium*, *Oxalobacter*, *Peptostreptococcus* and *Aggregabacter* were observed to be inversely associated with chemotherapy responses and with a decrease in progression-free survival in non-responders. Moreover, the abundance of *Ruminococcaceae* (documented to be related to vascular invasion) was significantly increased in the non-responder group [86].

### 2.4. Specific Microbial Features as a Diagnostic and Predictive Markers for CCA

Based on emerging findings, the differences in gut microbiota composition observed in CCA patients suggest that specific microbial features could serve as a non-invasive diagnostic tool for early CCA detection [87]. However, establishing causality remains challenging due to limited clinical trial follow-ups and potential confounding factors in observational studies. Chen et al. conducted a two-sample Mendelian randomization study to investigate potential causal associations between gut microbiota and CCA. By leveraging genetic variants, they identified 15 microbial taxa that may exert either protective or detrimental effects on CCA risk (higher abundance of the genera *Eubacterium nodatum* group, *Ruminococcus torques* group, *Coprococcus*, *Dorea* and phylum *Actinobacteria* were associated with reduced risk of eCCA, while higher abundance of the family *Veillonellaceae*, genus *Alistipes*, order *Enterobacteriales* and phylum *Firmicutes* were associated with increased iCCA risk; protective effects against CCA were suggested for the genera *Collinsella*, *Eisenbergiella*, *Anaerostipes*, *Paraprevotella*, *Parasutterella* and phylum *Verrucomicrobia*) [83]. Similarly, another study suggested potential associations between genetically predicted increases in *Veillonellaceae*, *Alistipes*, *Enterobacteriales* and *Firmicutes* with higher iCCA risk, whereas increases in *Anaerostipes*, *Paraprevotella*, *Parasutterella* and *Verrucomicrobia* appeared to have protective effects [88]. These findings support a potential causal link between distinct gut microbial signatures and iCCA risk.

Despite the observed changes and potential causal link in the microbiota of patients with CCA, establishing specific gut microbial changes for early CCA detection remains challenging. This difficulty arises from the dynamic nature of the microbiota and its constant fluctuations, making it hard to define a uniform group of healthy controls, as individual microbiomes vary significantly. Nevertheless, in recent years, ongoing research has increasingly focused on the development of diagnostic models based on specific microbial signatures of gut microbiota to predict the early diagnosis of CCA [89]. In a case–control study comparing 53 CCA patients, 47 patients with cholelithiasis and 40 healthy controls, researchers explored a gut microbiota model involving *Burkholderia*-*Caballeronia*-*Paraburkholderia*, *Faecalibacterium* and *Ruminococcus_1* for early CCA diagnosis. The study identified eight enriched genera in the cholelithiasis group (*Streptococcus*, *Agathobacter*, *Bifidobacterium*, *Ruminococcus gnavus* group, *Faecalibacterium*, *Subdoligranulum*, *Collinsella* and *Escherichia-Shigella*) and four enriched genera in the CCA group (*Bacteroides*, *Muribaculum*, *Muribaculaceae*_unclassified and *Alistipes*), highlighting a significantly different microbial community composition between groups (*p* = 0.001). These results suggest a progressive shift in gut microbiota composition from benign to malignant hepatobiliary diseases and ultimately to healthy controls [90]. Similarly, Deng et al. proposed a gut-microbiota-based diagnostic model incorporating eight key genera (*Faecalibacterium*, *Klebsiella*, *Lactobacillus*, *Dorea*, *Veillonella*, *Ruminococcus gnavus* group, *Burkholderia-Caballeronia-Paraburkholderia* and *Citrobacter*), which demonstrated high accuracy in distinguishing patients with CCA or HCC from healthy individuals. Interestingly, CCA patients exhibited higher alpha diversity compared to HCC patients [80]. Jia et al. identified a positive correlation between the genera *Lactobacillus* and *Alloscardovia* and the plasma–stool ratios of tauroursodeoxycholic acid (TUDCA). The combination of these two genera enabled discrimination between iCCA and other conditions, such as HCC, hepatic cirrhosis or healthy states. Patients with iCCA and vascular invasion had a higher abundance of the *Ruminococcaceae* family, elevated levels of interleukin (IL)-4 and increased levels of six conjugated bile acids. In contrast, they exhibited lower levels of IL-6 and chenodeoxycholic acid compared to patients without vascular invasion. This study identified a panel of biomarkers, including gut microbiota, bile acids and cytokines, for iCCA diagnosis and vascular invasion risk prediction [81]. Finally, a comparative study between CCA patients and healthy controls revealed a decreased *Firmicutes*/*Bacteroidota* (F/B) ratio in CCA patients. Notably, the abundance of *Klebsiella* was significantly higher, while *Bifidobacterium* levels were markedly reduced, leading to a decreased *Bifidobacterium*/*Klebsiella* (B/K) ratio. These findings suggest that *Klebsiella* and *Bifidobacterium* could serve as non-invasive intestinal biomarkers to enhance CCA diagnosis [91].

Recent studies suggest that specific gut microbiota models may be valuable for the early detection of CCA and for differentiating it from other conditions, such as benign biliary diseases and other types of cancer. However, large-scale cohort studies with more robust sample sizes are needed to analyze the microbiome using a multi-omics approach and to validate these preliminary findings. Additionally, the use of advanced cell culture systems, such as organoids and organoid co-cultures, may provide deeper insights into the complex interactions between the tumor microenvironment and gut microbiota components [92]. Confirming the potential role of gut microbiota in CCA could open new avenues for non-invasive cancer diagnosis.

### 2.5. Modulating Gut Microbiota in CCA

There are currently many questions regarding the interaction between the human microbiome and CCA. While the existing literature suggests a link between gut microbiota and CCA, the current lack of conclusive evidence prevents the establishment of a definitive cause-and-effect relationship. As a result, no formal recommendations can be made for assessing gut microbiota in CCA patients. Knowledge of the potential role of the microbiota in the microenvironment of this cancer may help to develop new diagnostic, prognostic or even therapeutic strategies. In addition, understanding the link between the gut microbiome and the liver may play an important role in enhancing responses to CCA therapies. From a therapeutic perspective, microbiota modulation holds promise for CCA prevention and treatment using probiotics or other therapies that alter the pathogenic microbiota portion and indirectly reduce inflammation and consequently the risk of developing CCA or slowing down its progression. Some randomized trials have tested probiotics in hepatobiliary tumors and have shown an increase in TNFα and IL1β in the postoperative period, supporting the use of probiotics to modulate the immune response and reduce infectious complications in patients. However, there is no evidence to support the modulation of gut microbiota in CCA patients through probiotics [93]. Even if fecal microbiota transplantation is a potential therapeutic strategy to restore eubiosis in several gastrointestinal diseases, there are no clinical trials evaluating the benefits of FMT on gut microbiota modulation in CCA. Given the improvement in CCA research, efforts should be made to develop new therapeutic strategies that modify the composition of the microbiota, targeting the main species altered in the case of CCA.

## 3. Non-Biological Approaches to the Diagnosis and Prognosis of CCA

### 3.1. Endoscopic Diagnosis of CCA

Abnormalities of the biliary tree can be studied using contrast media, either percutaneously or endoscopically. These cholangiography approaches are ERCP and percutaneous transhepatic cholangiography (PTC) [23]. PTC was first reported in 1983 with diagnostic and therapeutic indications [30]. The main indications include unreachable papilla, failed papilla cannulation (e.g., in the case of intradiverticular papilla) or difficult bile stones. Unfortunately, brush cytology and biopsy performed during ERCP or PTC have low diagnostic sensitivity in CCA patients [4,30]. The combination of brushing and biopsy has a diagnostic sensitivity of 70.7% [6]. This value rises to 88% when positive brush cytology is associated with elevated Ca 19-9 levels [6]. Improved diagnostic value comes from cholangioscopy. This application allows a direct visualization of the biliary lumen and mucosa [30,35]. It is therefore associated with an increased accuracy of malignancy by performing targeted biopsies [4]. Specifically, the direct optical visualization of CCA that can be obtained during cholangioscopy consists of the diagnosis of abnormal vessels, nodulations and irregular surface of the biliary epithelium with ulcerations. According to the morphological abnormalities documented during cholangioscopy, a modern classification of the mucosal pattern of biliary strictures according to the Mendoza criteria has been proposed [30]. Peroral cholangioscopy (POC) is superior to brush cytology [30]. A lot of evidence suggests that visual impressions during POC are more accurate and sometimes might be superior to a targeted biopsy; this finding is defined as the so-called “cholangioscopy paradox”. Cholangioscopy-guided biopsies have a sensitivity and specificity of 74% and 98%, respectively [30]. POC is an endoscopic technique introduced in the 1970s for the direct visualization of the biliary tree [30]. Its indication has increased over time with the introduction of newer technological boundaries such as narrow-band imaging (NBI), digital imaging analysis and FISH [35].

**Figure 2:**
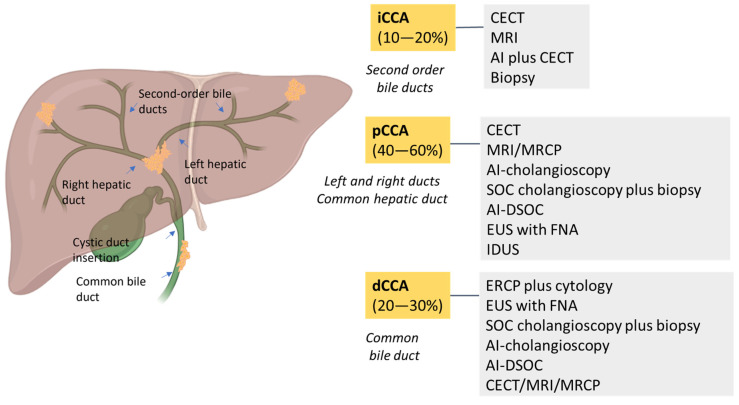
A schematic overview of the prevalence of CCAs and the best diagnostic methods. CCA is classified as intrahepatic CCA (iCCA) and extrahepatic CCA, which is divided into perihilar CCA (pCCA) and distal CCA (dCCA). iCCA is located proximally to second-order bile ducts and the insertion of the cystic duct into the common bile duct. dCCA is confined to the common bile duct below the cystic duct insertion.

Compared to conventional fluoroscopy-based ERCP, NBI is a useful tool in defining distal tumor margins in PSC patients with CCA without showing an increased detection of BilIN [35]. In 2007, a cholangioscope with fiberoptic scope was introduced, and in 2015, a digital version was developed with higher resolution in image quality and field of view. Currently, the cost of the device is the main critical point. All endoscopic modalities for CCA diagnosis have advantages and limitations (Figure 2).

ERCP has the advantage of visualizing and delineating the biliary stricture to facilitate biopsy or stenting but has the disadvantage of being invasive. Conversely, EUS has the advantage of providing a detailed examination of the biliary structure and the surrounding tissue with a rare risk of tumor seeding along the needle tract. Confocal laser endomicroscopy (CLE) is an innovative diagnostic method with real-time in vivo histological data during the endoscopic visualization of the biliary epithelium. The use of CLE on cholangioscopy was first reported in 2008 [30]. In 2022, the first study of AI applied to cholangioscopy was published. The AI model had an accuracy of 94%, a sensitivity of 94% and a specificity of 92% [30] (Table 2).

Biopsy is the only way to reach definitive diagnosis of CCA [3]. Biopsy may be contraindicated in the presence of ascites or severe coagulopathy [3]. Texture and color enhancement imaging (TXI) and red dichromatic imaging (RDI) are new approaches reported for the diagnosis of the CCA [94]. The novel application in endoscopy, which highlights slight differences in the color tone, improves the visibility of deep blood vessels and the structural changes in the biliary epithelium, makes the diagnosis of CCA more accurate [94].

### 3.2. Radiomics and Radiogenomics in the Diagnosis of CCA

In the era of precision medicine, research on data imaging analysis has led to the development of computational models that can support clinical decision-making processes, particularly in oncology [95]. Radiomics is an emerging field of medical imaging that enables the extraction of quantitative phenotypic data by exploiting the hypothesis that medical images reflect the biological behavior and histological architecture of the tissue included in the volume of interest (VOI) [96].

**Figure 3:**
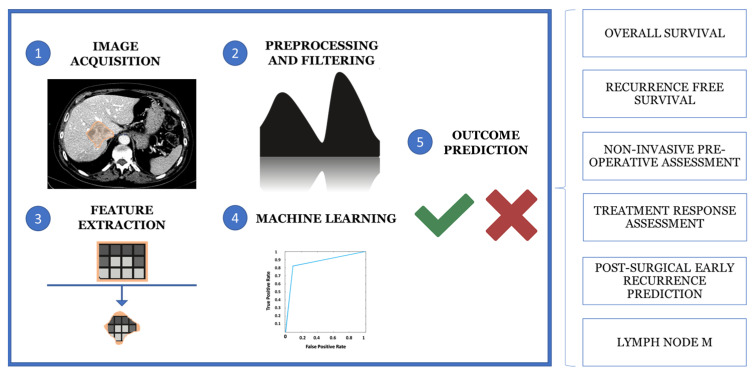
The multistep process of medical imaging analysis is based on image acquisition and reprocessing, segmentation, feature extraction, data analysis and model building.

Computational analysis of the VOI allows the extraction of quantitative and qualitative hallmarks, termed radiomics features, such as first-order statistics, texture-based metrics and geometric properties, to build predictive models. Moreover, radiomics and AI can be used together to extract and analyze quantitative features from medical images to support diagnosis, staging and treatment prediction using machine learning (ML) algorithms. This multi-step process involves five main phases: image acquisition and preprocessing, segmentation, feature extraction, data analysis and model building (Figure 3) [97,98,99].

The application of radiomic models has attracted considerable interest in the study of hepatic neoplasms, particularly the iCCA, due to their potential for more accurate patient risk stratification and disease categorization. Research topics include prediction of neoplastic disease outcome, in terms of overall or recurrence-free survival, non-invasive preoperative histological classification, response prediction to medical therapies (particularly biologicals), prediction of early postoperative recurrence, risk assessment of lymph node metastases and identification of novel disease biomarkers [100]. Differential diagnosis with other focal liver lesions, including HCC, is particularly challenging in poorly differentiated forms, where dynamic imaging, arterial hypervascularization and washout alone may affect the diagnostic accuracy. In this context, Liu et al. combined first- and second-order radiomic features extracted from T2-weighted fat-sat images, post-contrast MR arterial, portal venous and delayed phases of dynamic imaging with clinical parameters (e.g., CEA, CA 19-9, AFP, age, sex and vascular factors) to develop a robust hybrid radiomic with an AUC = 0.877 in differentiating ICC from HCC in the validation set [101]. Similar results were observed by Zhang et al. when combining an MR-based radiomic score with clinical radiological factors [102].

The preoperative heterogeneity of CCA poses a significant challenge in the differential diagnosis of hepatic abscess. Overlapping radiological features between iCCA and hepatic abscess may affect preoperative diagnosis and delay diagnosis. In this context, CT-based radiomic models and texture analysis showed promising results, achieving high specificity and sensitivity (81% and 94%, respectively) in trained datasets using arterial and portal phases of dynamic imaging [103]. The identification and the prediction of early recurrence in CCA is a hot topic in the literature, as it plays a pivotal role in surgical and therapeutic decisions. Clinical data are of limited value in preoperative risk stratification for recurrence [100]. A hybrid model combining CT radiomic signature, including peritumoral tissue characteristics, and clinical data showed a C-index of 0.71 in predicting early recurrence in patients with mass-forming CCA [104].

In this specific context, a clinical–radiological–radiomic nomogram forecasted early post-surgical recurrence with an accuracy comparable to post-surgical scoring systems incorporating histopathology. Similarly, radiomic models based on MR imaging, in particular dynamic post-contrast sequences and pre-contrast T1-weighted images, combined with patient clinical data, allowed accurate prediction of microvascular invasion (MVI) in iCCA mixed hepatoma. This approach demonstrated superiority over standalone clinical or radiomic models and provided clear stratification of survival outcomes [105]. As MVI is associated with worse prognosis and poor surgical outcomes, preoperative non-invasive prediction of MVI could guide earlier decisions for more extensive surgical resections to reduce recurrence or incorporate neoadjuvant chemotherapy.

Lymph node metastasis (LNM) is an independent risk factor for mortality and is strongly correlated with overall survival. Currently, CT, MR and functional PET-CT imaging have limitations in identifying LNM, often relying primarily on qualitative features (e.g., size, margins, inhomogeneous signal intensity and increased F-FDG uptake). Radiomics models integrating clinical data with MR-derived features, including DWI and ADC maps, achieved an accuracy of 0.90 in detecting LNM [106]. Such models have been proposed to guide imaging intervals, initial therapeutic strategies and preoperative management. Similarly, Xu et al. reported an AUC of 0.87 when using a T1-weighted contrast-enhanced MR-based support vector machine (SVM) in combination with CA 19-9 levels [107].

Research on immunotherapy response assessment has recently focused on tumor-associated tertiary lymphoid structures (TLSs), which have been positively correlated with favorable prognosis and good relapse-free survival [108,109]. A Chinese study reported a correlation between the presence of TLS and improved recurrence-free survival in patients with low-risk RM-derived radiomic scores with significant differences. These results strongly suggest that TLS-related radiomic models serve as biomarkers for predicting responses to biological therapies [110]. Similar radiomics models constructed from a combination of first- and second-order features related to tumor heterogeneity (e.g., grey-level-related features and texture heterogeneity) and clinical variables have shown better performance than clinical data or imaging alone in predicting disease-free survival in the same study, demonstrating superiority of radiomics over current clinical scores [110]. The integration of radiomics with other “-omic” disciplines has recently promoted the development of hybrid models that integrate imaging with molecular, histological and genetic pathways for a more precise subcategorization of hepatic neoplasms (e.g., radiometabolomics and radiogenomics). For instance, Sadot and co-workers demonstrated correlations between radiomic features and EGFR expression as well as hypoxia factors, paving the way for non-invasive molecular profiling of iCCA [111].

### 3.3. Artificial Intelligence in Diagnosis of CCA

Biomedicine is strongly influenced by AI techniques, and this is particularly true when oncological disciplines are considered [112]. AI is defined as the ability of a computer or a robot to perform tasks that are associated with human intelligence [17]. AI includes logistic regression (LR) models, artificial neural networks (ANNs), support vector machines (SVMs) and convolutional neural networks (CNNs) [20,113]. The AI consists of ML techniques and natural language processing methods [114]. The peculiar advantage of AI is its ability to analyze large amounts of clinical data and identify specific disease patterns [115]. Currently, the huge number of radiological images, coupled with clinical data, has led to the expansion of AI in the field of radiology and radiomics [100,116]. In the early diagnosis of CCA, AI is able to identify the histological, cytological or radiological aspects and correctly extract CCA-specific features [117]. The application of AI in the diagnosis and treatment of CCA is gradually increasing [118,119]. Differential diagnosis of CCA requires experience and knowledge due to the variety of radiological presentations. Interestingly, AI recognizes complex patterns in imaging data and uses specific automatisms to provide a quantitative and objective assessment of tumors [120]. Using a neural network model, clinicians were able to classify the metabolomic and proteomic profiles of bile from CCA patients by increasing the accuracy of differential diagnosis with benign biliary strictures [121]. A popular ANN, the multi-layer perceptron (MLP), is used for decision making to differentiate images with CCA [122]. The AI-MLP showed an accuracy of 88%. Two diagnostic tools applied to cholangioscopy are CLE, a new technology that employs a low-energy laser light emission to generate tissue images with enhanced biopsy precision, and AI as described [30,123]. In contrast to CLE, AI is gaining increasing attention for its endoscopic applications, considering its results in the endoscopic field of colonoscopy [124,125]. Endoscopy plays a pivotal role in diagnostic and therapeutic management of biliary diseases [126,127]. In particular, the cholangioscopy has a great performance in discriminating malignant conditions such as CCA from precancerous lesions or benign inflammatory diseases [125,128]. The AI in cholangioscopy has a recent application as demonstrated in a randomized controlled trial, where digital single-operator cholangioscopy (DSOC)-guided biopsy specimens showed superiority to ERCP-guided brushing (68.2% vs. 21.4%) in the diagnosis of malignant hilar stricture [129,130]. There is increasing evidence to support the role of AI in the diagnosis of CCA (Table 2).

**Table 2:** CCA diagnostic approaches in current clinical practice.

METHODS FOR CCA DIAGNOSIS	ACCURACY (%)	SENSITIVITY (%)	SPECIFICITY (%)	REFERENCES
BIOLOGICAL APPROACHES				
**Ca 19-9**		50–90	54–98	Kodali S et al., 2024 [3] Shin DW et al., 2023 [6]
**CEA (>5.2 ng/mL)**		68	82	Shin DW et al., 2023 [6]
**FISH**		70	60–97	Ilyas SI et al., 2018 [24] Azeem N et al., 2014 [35]
**Brush cytology**		27–56		Dar FS et al., 2024 [13]
**Brush cytology plus biopsy and elevated CA 19.9**		70.7–88	97	Doong Woo Shin et al., 2023 [6]
**Brush cytology plus biopsy and FISH**		70.7–82		Dar FS et al., 2024 [13]
**Brush cytology and elevated CA 19.9**		88	97	Shin DW et al., 2023 [6]
***NON-BIOLOGICAL APPROACHES***				
**AI-cholangioscopy (eCCA)**	89–95	81–94.7	91–92.1	Njei B et al., 2023 [20] Saraiva MM et al., 2022 [125]
**SOC cholangioscopy plus biopsy**	75–82	65–72	98–99	Njei B et al., 2023 [20] Dar FS et al., 2024 [13] Azeem N et al., 2014 [35]
**PTC plus cholangioscopy**	80–92			Doong Woo Shin et al., 2023 [6]
**AI plus CECT (iCCA)**	80.4–84.6			Njei B et al., 2023 [20]
**MRI/MRCP**		88–90	75–85	Ilyas SI et al., 2018 [24] Rushbrook SM et al., 2024 [4]
**CECT**	86	75–89	79–80	Ilyas SI et al., 2018 [24] Rushbrook SM et al., 2024 [4]
**EUS with FNA (dCCA)**		76–81	100	Ilyas SI et al., 2018 [24] Dar FS et al., 2024 [13]
**ERCP plus cytology**		21–70	20–100	Ilyas SI et al., 2018 [24] Azeem N et al., 2014 [35] Kodali S et al., 2024 [3] Rushbrook SM et al., 2024 [4]
**EUS with FNA (eCCA)**		43–89		Ilyas SI et al., 2018 [24] Shin DW et al., 2023 [6]
**IDUS**	91	93	89.5	Ilyas SI et al., 2018 [24]
**SOC plus visual impression**		78–90	71.2–82	Ilyas SI et al., 2018 [24] Mauro A et al., 2023 [30] Rushbrook SM et al., 2024 [4]
**SOC plus biopsy**		66	97	Ilyas SI et al., 2018 [24]
**DSOC**		94	95	Mauro A et al., 2023 [30]
**DSOC plus biopsy**		74	98	Mauro A et al., 2023 [30]
**AI-DSOC**	94.9	94.7	92.1	Mauro A et al., 2023 [30]

Biological approaches: carcinoembryonic antigen (CEA); carbohydrate antigen 19-9 (CA 19-9); fluorescence in situ hybridization (FISH). Non-biological approaches: artificial intelligence (AI); contrast-enhanced computer tomography (CECT); digital single-operator cholangioscopy (DSOC); endoscopic ultrasound (EUS); endoscopic retrograde cholangiopancreatology (ERCP); fine-needle aspiration (FNA); intraductal ultrasound (IDUS); magnetic resonance imaging (MRI); magnetic resonance cholangiopancreatography (MRCP); percutaneous transhepatic cholangiography (PTC); single-operator cholangioscopy (SOC).

Histology remains the gold standard for the diagnosis of malignancies including CCA, but AI assistance in histology has not always shown a realistic benefit [54]. It is recognized that AI has an excellent accuracy rate in distinguishing HCC from CCA using a DL model, but AI assistance may not improve the performance of pathologists. Moreover, preoperative variables included in AI models could identify patients at risk of “futile” surgery, defined as disease recurrence or death within 12 months after surgical CCA resection [131]. By imputing the preoperative clinicopathologic parameters of the CCA patient, it is possible to estimate the predicted probable “futile” outcome considering any combination of radiologic TBS and serum CA 19-9 levels. Following resection of iCCA, 50–70% of patients will experience a recurrence or a very early recurrence, defined as recurrence within five months, which is associated with a poor prognosis. The use of different ML and DL techniques could identify patients at high risk for “futile” surgery for iCCA [104,132,133,134,135]. In addition, a multi-omics approach, including genomics, radiomics and pathomics, combined with AI algorithms, will have the potential to achieve favorable outcomes through its “super-model” prediction of neoplasia [136,137]. As reported in the scientific literature, AI models have the ability to improve the accuracy of the diagnosis, treatment and prognosis of CCA [1]. CECT and MRI integrated with 18F-DG-PET CT are valuable tools harnessed by AI to anatomically define CCA margins [1]. A serologic model using AI technologies was developed for CCA diagnosis. By analyzing 15 serum bile acids, it was able to diagnose CCA with a sensitivity of 79% and an accuracy and specificity of 86.4%, and 100%, respectively. In addition, an ANN-based model using a combination of CA19-9 and alkaline phosphatase showed promise in diagnosing CCA with a sensitivity and specificity of more than 95% [1]. Regarding the role of cytology obtained after ERCP brushing, the use of a neural network model studying the metabolomic and proteomic profile of bile from CCA patients was able to distinguish CCA from benign lesions with a sensitivity and specificity of 94.1% and 92.3%, respectively [1]. The results of epithelial brushing are classified as non-diagnostic, negative for malignancy, atypical, neoplastic, suspicious for malignancy or malignant [4]. In 2021, a CNN model was developed to diagnose CCA from microscopic hyperspectral pathology slides. This model was able to diagnose CCA with 88.3% accuracy [1]. CNN models based on the combination of CECT with serum biomarkers (CEA and CA 19-9) have shown superiority in the detection of CCA (0.68 vs. 0.45; *p* = 0.04) [1]. Using AI technologies, it is possible to differentiate CCA from HCC with 88% accuracy. In addition, AI models allow clinicians to differentiate between high- and low-risk CCA patients and LNM [1]. AI has a documented role in predicting CCA prognosis by identifying a group of otherwise undetectable high-risk patients who may benefit from neoadjuvant treatment prior to surgical resection as they have a high recurrence rate. The clinical value of US in CCA is limited and depends on individual expertise [5]. On US, CCA appears as an intrahepatic hypoechoic lesion with biliary duct dilatation. An AI-assisted US is a great tool to increase the accuracy of CCA diagnosis. A recent AI model (YOLOv5) has shown an increased accuracy of 13% in the classification of benign and malignant focal liver lesions [5]. Although US screening for CCA is challenging, the YOLOv5 model demonstrated excellent performance in classifying CCA lesions with a diagnostic sensitivity and specificity of 96.51% and 97.85%, respectively [5]. Further research is needed to validate AI models in real clinical practice to determine their utility and acceptability as adjunctive tools in CCA patients.

## 4. Conclusions and Future Perspectives

**Figure 4:**
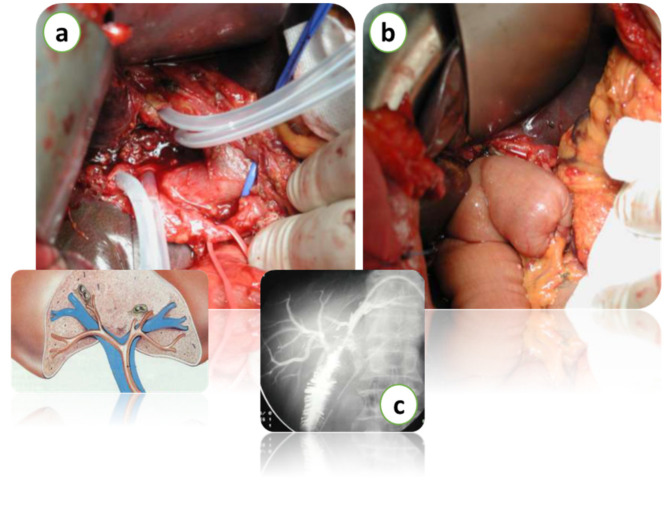
Intraoperative images of hepatectomy for Klatskin’s tumor with biliary resection over the 2nd confluence (**a**). Bilio-enteric reconstruction (**b**). Intraoperative radiological imaging of bilio-enteric anastomosis (**c**). Courtesy of Professor Paolo Innocenti, from his personal archive.

Over the past decade, new approaches have been validated to improve visualization of the biliary tree and its morphologic abnormalities. In CCA patients, the initial imaging workup is based on a cross-sectional imaging study with CECT or MRI and MRCP [13]. The use of quantitative imaging and a colorful and well-visualized 3D analysis system has increased the accuracy of biliary strictures [24]. The integration of radiomics with other “-omic” disciplines has recently promoted the development of hybrid models that integrate imaging with molecular, histological and genetic pathways for a more accurate subcategorization of hepatic neoplasms. POC is a valid tool that improves the diagnostic accuracy of CCA and influences surgical decision marking by complementing the limited sensitivity of current imaging modalities and ERCP procedures. POC implements the diagnostic performance of ERCP by showing high sensitivity for visual findings [19]. The results of POC in preoperative and staging of CCA could be improved by integrating the promising approaches such as the narrow-band imaging or texture and color enhancement imaging or red dichromatic imaging. Standard treatments for CCA include surgery, chemotherapy and radiotherapy, radiofrequency ablation, photodynamic therapy and immunotherapies, together with liver transplantation in selected cases (Figure 4).

Although surgery with curative intent remains the cornerstone of treatment, recurrence and metastatic progression underline the need for comprehensive multidisciplinary therapeutic care [16]. The resectability of CCA is based on the presence of metastases, on patient and local factors. There is no consensus on the indication for surgery based on local factors. In the experience of the Memorial Sloan Kettering Cancer Centre, the prediction of resectability is based on the assessment of vascular invasion and liver atrophy. Currently, the criteria that render CCA unresectable are the following: involvement of the hepatic duct up to the secondary biliary ducts, bilaterally; encasement or occlusion of the portal vein associated with or without atrophy of the contralateral hepatic lobe; and involvement of the hepatic duct up to the secondary biliary ducts with atrophy of the contralateral hepatic lobe [16]. Molecular perturbation analysis and precision oncology in CCA are the milestones of future hepatobiliary research, and this should be strongly supported by clinicians and developed in the form of guidelines. Coordinated efforts are needed in understanding CCA and translating the basic research into clinical practice. There is a need to develop and implement bioinformatics applications for the diagnosis of CCA. Multidisciplinary collaboration is a crucial aspect for the development of precision medicine in CCA patients. The future perspectives of precision medicine are based on translational research, knowledge-based informatics models and understanding the progression by exploring the molecular features of CCA. This integration of gene expression and proteomics data with the clinical imaging features will provide a better way to achieve a more accurate diagnosis of CCA. The liver and bile ducts have a close bidirectional relationship with the gut microbiota. Recent findings suggest that the gut microbiota may not only have an influence on the progression of CCA but also on the response to drug therapies. Therefore, a promising approach that could improve treatment strategies for CCA would be a deeper understanding of the microbiota and its role in promoting chronic inflammatory stimuli. Many AI tools have been used to diagnose CCA, with their respective advantages and limitations. The use of AI in the diagnosis and treatment of CCA is gradually increasing. Preoperative variables analyzed and included AI models could identify patients at risk of “futile” surgery for CCA. Novel preclinical and clinical research models are urgently designed to potentially identify factors that influence the development and progression of human CCA.

Early and accurate diagnosis of CCA remains a major challenge. There are ongoing efforts to identify and validate protein biomarkers and AI models for the diagnosis, prediction and prognosis of CCA. Human decision making and judgement are still needed to solve CCA together with the development of novel AI algorithms and precision medicine. Considering the improvement in CCA research, efforts should be made to advance the involvement of the microbiota in CCA progression, targeting the major species altered in hepatobiliary diseases. The improvement of precision therapy in the treatment of CCA is strongly advocated. The challenge ahead will be to develop innovative and personalized treatment of care according to the validation of combined diagnostic protocols. The current variability in CCA guidelines and recommendations should be reconsidered and discussed towards a more standardized diagnostic and therapeutic work-up.

## Figures and Tables

**Table 1:** Microbial signatures in CCA patients.

Microbiota	Perihilar CCA	Intrahepatic CCA	Extrahepatic CCA
**Bile**	*E. faecalis* *E. faecium* *E. cloacae* *E. coli* *Pyramidobacter* *Klebsiella* *Bacteroides* *Enterococcus*	*Firmicutes* *Bacteroidetes* *Actinobacteria* *Verrucomicrobia*	*Bacteroides* *Geobacillus* *Meiothermus* *Anoxybacillus*
**Blood**	*Ruminococcaceae* *Oribacterium* *Oxalobacter* *Peptostreptococcus* *Aggregabacter* *Burkholderia* *Caballeronia* *Paraburkholderia* *Faecalibacterium*		

## Data Availability

Data are contained within the article.
